# Behaviour of the Foramen Ovale Flow in Fetuses with Intrauterine Growth Restriction

**DOI:** 10.1155/2018/1496903

**Published:** 2018-01-17

**Authors:** Ângela R. L. Nader, Paulo Zielinsky, Alexandre Antonio Naujorks, Luiz Henrique S. Nicoloso, Antonio Luiz Piccoli Junior, Natássia Miranda Sulis, Luiza Ferreira van der Sand, Victoria de Bittencourt Antunes, Gabriela dos Santos Marinho, Fernanda Greinert dos Santos, Natan Pereira Gosmann, Eduardo Becker Júnior, Renato Frajndlich, Tamara Beherens, Marcelo Brandão da Silva, Caroline Barbisan, Stefano Busato, Mauro Lopes, Caroline Klein

**Affiliations:** ^1^Unidade de Cardiologia Fetal, Instituto de Cardiologia do Rio Grande do Sul, Porto Alegre, RS, Brazil; ^2^Departmento de Pediatria, Universidade Federal do Rio Grande do Sul (UFRGS), Porto Alegre, RS, Brazil; ^3^Hospital de Clinicas de Porto Alegre, Porto Alegre, RS, Brazil

## Abstract

**Background:**

Foramen ovale (FO) flow may be altered in IUGR. This study was designed to test this hypothesis.

**Methods:**

Forty pregnant women (24–38 weeks) were divided into 3 groups: group I (IUGR), group II (adequate growth and maternal hypertension), and group III (normal controls). Impedance across the FO was assessed by the FO pulsatility index (FOPI): (systolic velocity − presystolic velocity)/mean velocity. Statistical analysis utilized ANOVA, Tukey test, and ROC curves.

**Results:**

Mean FOPI in IUGR fetuses (*n* = 15) was 3.70 ± 0.99 (3.15–4.26); in the group II (*n* = 12), it was 2.84 ± 0.69 (2.40–3.28), and in the group III (*n* = 13), it was 2.77 ± 0.44 (2.50–3.04) (*p*=0.004). FOPI and UtA RI were correlated (*r* = 0.375, *p*=0.017), as well as FOPI and UA RI (*r* = 0.356, *p*=0.024) and, inversely, FOPI and MCA RI (*r* = −0.359, *p*=0.023).

**Conclusions:**

The FO flow pulsatility index is increased in fetuses with IUGR, probably as a result of impaired left ventricular diastolic function.

## 1. Introduction

Intrauterine growth restriction (IUGR) is a significant clinical problem, affecting up to 10% of all pregnancies [1] and even 15% of all monochorionic twin pregnancies [2], with high perinatal morbidity and mortality rates due to fetal hypoxia [3, 4]. Placental insufficiency is the etiology in most cases [5, 6], although infections, congenital anomalies, and drug misuse are other associated conditions [7]. Monitoring the consequences of fetal hypoxia is the basic obstetric management, given that the only current treatment for IUGR is delivery [7, 8]. The fetal heart is a central organ in adaptive mechanisms to hypoxia, and cardiac dysfunction is recognized as the pathophysiologic determinant of clinical deterioration in both early- and late-onset IUGR [4]. Biophysical profile and ductus venosus impedance become abnormal only in advanced stages of fetal compromise [7], and for this reason, alternative parameters, in particular cardiac function, could provide earlier markers with higher sensitivity [7, 9].

In normal pregnancy, a progressive decrease in umbilical artery (UA) impedance occurs, which allows appropriate fetal cardiovascular development [4]. IUGR fetuses have abnormal placental changes, with increased placental vascular resistance and progressive deterioration of the UA flow [10]. UA Doppler is indicated for early detection of placental insufficiency [11]. Fetal circulatory response primarily benefits the systemic cardiac output (CO), providing an adequate oxygen supply to vital organs [12, 13]. With progression of fetal pulmonary and systemic vasoconstriction, an increased right ventricular afterload and a shift of cardiac output to the left ventricle occur [8], with impact on left diastolic function and possible increased impedance to flow through the foramen ovale (FO) [14]. Due to its triphasic flow pattern, the vascular pulsatility index (PI) may represent its impedance, as already demonstrated in other situations such as gestational diabetes [15]. The analysis of the foramen ovale flow dynamics in fetuses with IUGR has not been previously assessed.

The purpose of this study was to compare the foramen ovale pulsatility index (FOPI) in IUGR fetuses with the FOPI in fetuses classified as of appropriate growth, with and without maternal hypertension. Correlations of the FOPI with maternal, fetal, and placental Doppler indices were tested.

## 2. Methods

A cross-sectional, controlled, nonblinded study of 40 single fetuses with 24 weeks of gestational age (GA) or more was designed. The sample included 15 growth-restricted fetuses (group I), 12 fetuses with normal weight for gestational age from hypertensive mothers (group II), and 13 fetuses with normal weight for gestational age from healthy mothers (group III). Maternal hypertension was classified according to Guidelines of the American College of Obstetricians and Gynecologists [16]. Fetuses with other abnormalities or fetuses whose mothers used drugs or tobacco were excluded from the study.

All pregnant women provided written informed consent to participate in the study, which was approved by the Medical Research Ethics Committee of the Institute of Cardiology of Rio Grande do Sul.

Gestational age was determined in all fetuses by first trimester ultrasound. After 24 weeks, a morphological ultrasound was performed for weight estimation (according to the Hadlock method) [17], placental assessment, amniotic fluid volume determination, and flow velocimetry of the umbilical, middle cerebral, and uterine arteries. Under fetal apnea, the resistance index (RI) was obtained by Doppler flow analysis and determined by the ratio (systolic velocity − diastolic velocity)/systolic velocity. The amniotic fluid index was measured by the sum of the pockets of the four quadrants of the maternal abdomen.

IUGR with placental insufficiency was defined by fetal weight below the 10th percentile for gestational age in combination with abnormal Doppler indices (either umbilical artery RI > 95th centile, middle cerebral artery RI < 5th centile, or uterine artery RI > 95th centile for gestational age).

**Figure 1:**
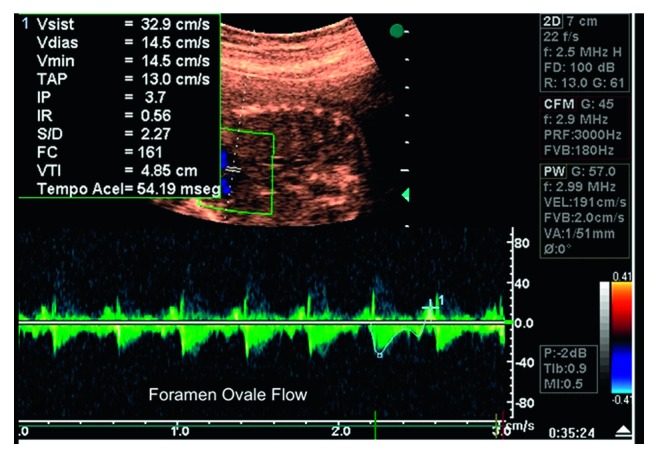
Foramen ovale flow and pulsatility index assessment: echocardiography of fetus at 33 weeks with intrauterine growth restriction. The foramen ovale pulsatility index is 3.70.

Fetal echocardiography was performed using an Acuson Aspen (Acuson, Mountain View, CA, USA) ultrasound system, with a multifrequencial transducer (3 to 5 MHz). Cardiac structural assessment used segmental sequential analysis. Flow in the FO was obtained at a four-chamber view, with the pulsed Doppler sample volume placed at the left atrial surface of the orifice in the central portion of the color flow mapping, with an angle of less than 20° [15, 18]. Flow impedance was assessed by the pulsatility index, obtained by the ratio (systolic velocity − presystolic velocity)/mean velocity [19] (Figure 1).

The statistical package SPSS version 15.0 (SPSS Inc., Chicago, IL) was used for data analyses. Quantitative analyses were reported as mean ± standard deviation (SD). Analysis of variance (ANOVA) was used for each variable. The Tukey test was applied for the individual assessment of each group when significant differences were present. The chi-square test of the observed frequencies was used for qualitative analyses. The correlation of the FOPI with fetal arteries' RI was tested by the Pearson test. The critical significance level was *p* < 0.05. The sample size was not calculated since no previous studies were available to assess differences. Nevertheless, the study power was calculated based on the pulsatility index of the foramen ovale, considering a significance level of 5%, standard deviations of 0.44 and 0.99, and a difference of 0.93 in the pulsatility indices of groups I and III, achieving a study power of 84%.

The reproducibility of measurements of the FOPI was tested. Intraobserver variability was assessed in twelve nonconsecutive normal fetuses by repeating the measurements on two occasions (2 days apart) with the same conditions. Interobserver variability was also assessed, with measurements repeated in 10 normal fetuses on the same day by a second observer blinded to the results of the first examination. Intraclass correlation coefficients were calculated to measure the degree of consistency among measurements. The Bland–Altman plots were created to show the mean of differences between measurements.

## 3. Results

Table 1 presents the characteristics of the sample, showing that the groups were comparable except for maternal age and body mass index (BMI) in group II when compared to the other two groups. Gestational age ranged from 24 to 38 weeks. Minimum maternal age was 14 years and maximum 38 years.

**Figure 2:**
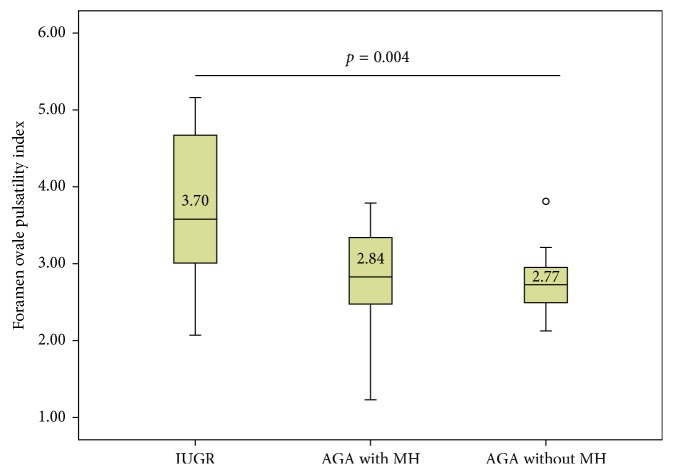
Mean of foramen ovale pulsatility index and *p* value for ANOVA between study groups. IUGR = intrauterine growth restriction; AGA = adequate for gestational age; MH = maternal hypertension.

Doppler features of study groups are shown in Table 2. The FOPI (mean ± standard deviation) in group I was 3.70 ± 0.99 (95% confidence interval: 3.15 to 4.26), 2.84 ± 0.69 (95% CI: 2.40 to 3.28) in group II, and 2.77 ± 0.44 (95% CI: 2.50 to 3.04) in group III (*p*=0.004) (Figure 2).

**Figure 3:**
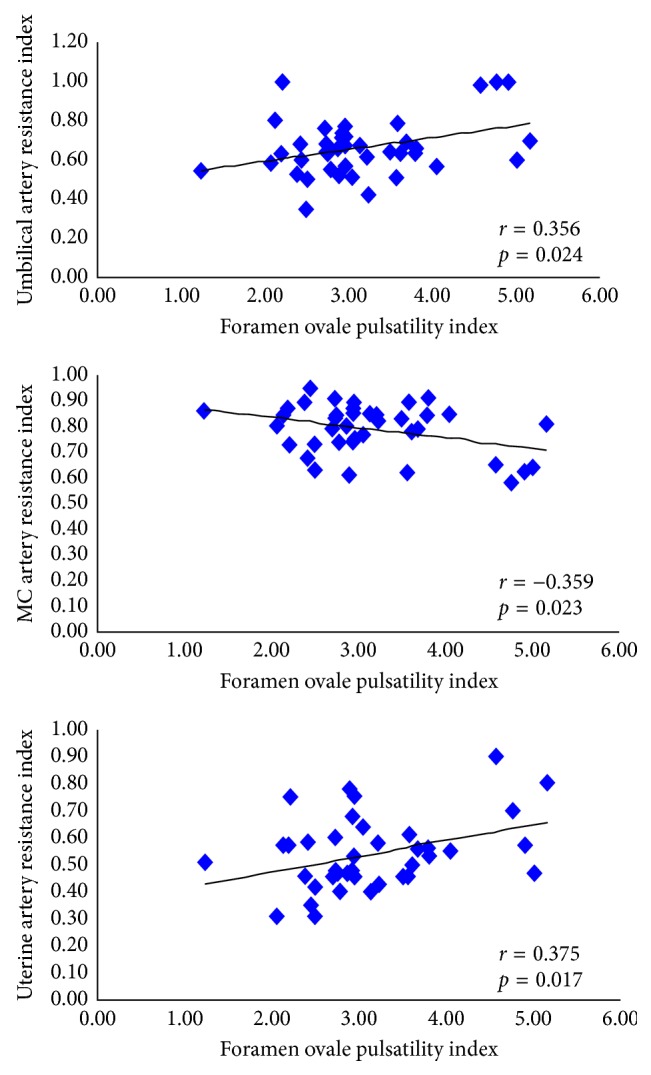
Pearson correlation of the foramen ovale pulsatility index with resistance index of umbilical, median cerebral, and uterine arteries. MC = median cerebral.

Pearson analysis of the FOPI showed a positive correlation of the FOPI with the UA (*r* = 0.356, *p*=0.024) and the UtA (*r* = 0.375, *p*=0.017). The correlation was negative with the MCA (*r* = −0.359, *p*=0.023) (Figure 3).

**Figure 4:**
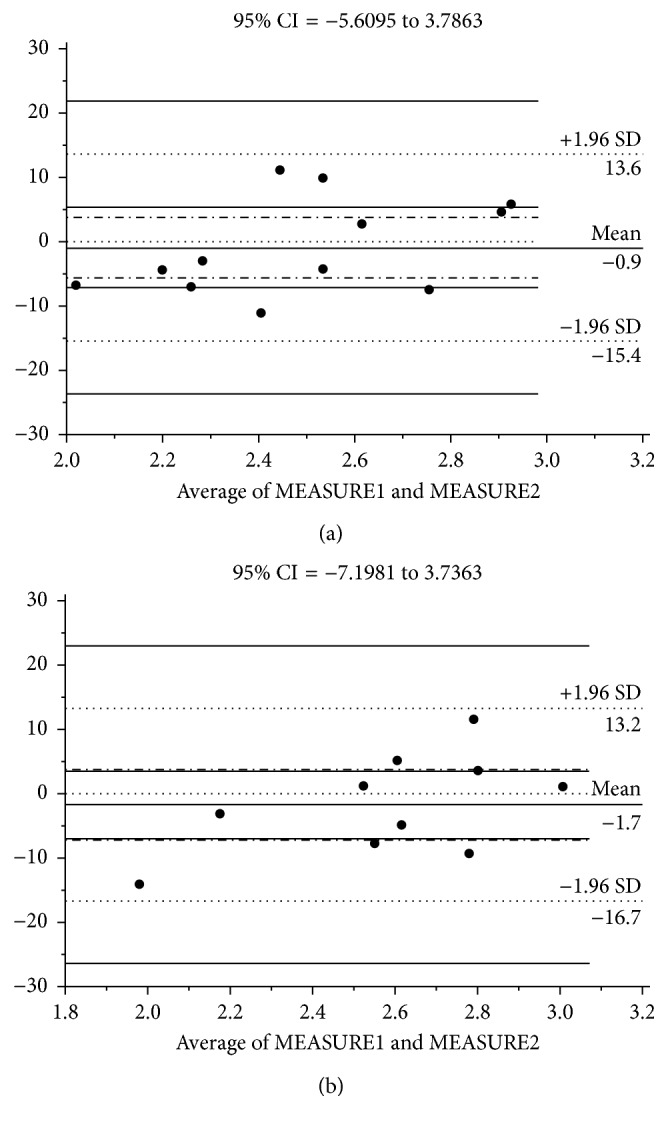
Bland–Altman plots showing intraobserver (a) and interobserver (b) variation in measurements of the foramen ovale pulsatility index. CI = confidence interval; SD = standard deviation.

To assess the agreement between two sets of measurements, intraclass correlation coefficients for FOPI measurements were estimated: intraobserver variation was 0.8035 (95% CI: 0.5002 to 0.9312) and interobserver variation was 0.8227 (95% CI: 0.4371 to 0.9528). The Bland–Altman plots were created (Figure 4).

## 4. Discussion

In this study, we assessed the foramen ovale flow dynamics in fetuses with IUGR and with adequate growth of both normotensive and hypertensive women. It was observed that IUGR fetuses had an increased impedance to flow through the FO, represented by an increased FOPI compared to control groups, probably as a result of impaired diastolic function. The same effect has been demonstrated in fetuses of diabetic mothers (FDMs) with myocardial hypertrophy, where the FOPI was higher than that in normal fetuses [15]. Several studies have demonstrated changes in diastolic function in FDMs, being the ductus venosus PI [20] and the pulmonary vein PI [21] significantly higher in fetuses with myocardial hypertrophy, probably due to a lower ventricular compliance. The mobility of the septum primum is lower in fetuses with IUGR [22] and myocardial hypertrophy [23] when compared to normal fetuses, due to a decreased ventricular compliance. The shortening fraction of the left atrium was also shown to be decreased in fetuses with myocardial hypertrophy, presenting a negative correlation with the septal thickness [24].

Left ventricular myocardial diastolic velocities by tissue Doppler imaging (TDI) were shown to be significantly higher in FDM, irrespective of the presence of cardiac hypertrophy, suggesting that maternal diabetes is associated with changes in left ventricle diastolic function even without myocardial hypertrophy [25]. TDI evaluation of IUGR fetuses showed that myocardial early and late diastolic velocity ratios were higher in IUGR in lateral and septal mitral annulus, when compared with normal growing fetuses [9]. A subsequent study [26] confirmed these findings. Both studies concluded that TDI can probably be a more sensitive method for detection of diastolic cardiac dysfunction in IUGR fetuses than conventional mitral and tricuspid valve Doppler [9, 26]. In a recent study in rabbits, cardiac weight reduction was demonstrated in response to IUGR, due to a decrease in the number of cardiomyocytes in both ventricles. However, an increase in the mean volume of cardiomyocytes occurred in the left ventricle. This demonstrates that the right and left ventricles respond differently to placental insufficiency [27].

IUGR fetuses have impaired ventricular filling, with higher atrioventricular valve E/A ratios, lower aortic and pulmonary artery systolic peak velocities, left ventricular CO increase, and right ventricular CO decrease. These hemodynamic changes favor flow into the left ventricle in order to maintain cerebral perfusion [7, 18]. Therefore, in the early stages of the disease, adequate levels of oxygen and substrates are maintained, despite the reduction in placental transfer. During the process of centralization, fetal changes occur in cardiac afterload, decreasing left ventricle afterload due to cerebral vasodilatation and increased systemic vasoconstriction [28]. Furthermore, hypoxia may impair myocardial contractility, while polycythemia may increase blood viscosity [7].

In fetuses with IUGR, a faster presystolic left-to-right flow velocity through the FO is in accordance with a study that assessed flow in fetuses with left ventricle hypoplasia or left side obstruction, in which there was increased left atrial pressure [29].

Umbilical and uterine artery flow RIs were not different between groups. Correlation analysis of the RI of maternal and fetal flows with the FOPI showed a positive weak correlation with the UA and the UtA and a negative weak correlation with the MCA. Nonstratification according to the severity of placental insufficiency in group I could be a limitation and may explain these data, as a stronger correlation would be expected with more severe fetal compromise. Left diastolic function assessed by the FOPI could already be altered in cases of IUGR with placental insufficiency in early stages. Turan et al. [28] assessed the time of onset of placental dysfunction in fetuses with IUGR, and in two of the three study groups, patients were recruited when there was no placental dysfunction. This situation was diagnosed only two to four weeks later. The fetal cardiac output is redistributed during hypoxia, promoting flow to vital organs like brain, heart, and adrenal glands [30–32] and justifying the MCA resistance decrease associated with cardiac diastolic dysfunction assessed by FOPI.

The possibility of maternal hypertension without IUGR is supported by other authors. Grisaru-Granovsky et al. [33] showed that the presence of IUGR in fetuses of hypertensive mothers was not correlated with worsening of the hypertensive disorder but reflected the individual predisposition of fetuses to abnormal development. Our study had a small sample size of group I, not allowing categorization in relation to the presence of associated maternal hypertensive disorder.

This study has limitations. It was not blind, allowing the potential occurrence of a mensuration bias. Being a cross-sectional study, it was not possible to assess the sequential changes in flow through the FO with the evolution of pregnancy. The lack of stratification according to the severity of fetal compromise and maternal hypertensive disorder in group I has already been mentioned. It was observed that group II had higher maternal ages and BMI than the other groups, but these are not expected to primarily interfere with fetal cardiac hemodynamics.

Despite the occurrence of oligohydramnios in one-third of the IUGR sample, technical aspects of assessment of the FOPI did not represent any limitation, as demonstrated by the strength of agreement between two sets of measurements obtained by trained operators.

It has been reported that IUGR fetuses may show different outcome patterns [28]. For this reason, it is important to perform serial tests in an effort to promote birth with the lowest possible morbidity and mortality. The use of other techniques, such as fetal echocardiography, may help monitoring IUGR fetuses [34]. The present study suggests that the presence of an increased FOPI, especially above 2.95, may suggest abnormalities in fetal diastolic heart function, thus potentially influencing the overall obstetric management.

## Figures and Tables

**Table 1:** Characteristics of the study groups. Maternal, fetal, and amniotic features are shown.

Variable	Group I (IUGR) (*n* = 15)	Group II (AGA with MH) (*n* = 12)	Group III (AGA without MH) (*n* = 13)	*p* value
Maternal age (years)	24.46 ± 4.56	31.08 ± 4.64	22.23 ± 5.64	<0.001^†^
Gestations	2.20 ± 1.14	2.42 ± 1.08	1.92 ± 1.18	0.560
Abortions	0.27 ± 0.59	0.08 ± 0.28	0.15 ± 0.55	0.640
Gestational age (weeks)	31.21 ± 3.91	31.80 ± 4.31	30.01 ± 3.96	0.529
Maternal weight gain (kg)	7.36 ± 3.41	9.93 ± 8.55	7.41 ± 2.92	0.399
Maternal body mass index	27.67 ± 6.10	34.73 ± 5.57	27.00 ± 4.30	0.001^∗^
Fetal weight (g)	1221.9 ± 584.6	1768.8 ± 773.9	1600.1 ± 626.7	<0.001^∗^
AFI^a^ < 8 cm	5 (33.3%)	2 (16.7%)	1 (7.7%)	<0.001^∗^

IUGR: intrauterine growth restriction; AGA: adequate for gestational age; MH: maternal hypertension; kg: kilograms; g: grams; AFI: amniotic fluid index; ^a^adjusted values for gestational age. ^†^*p* < 0.05 between group II and groups I and III; ^∗^*p* < 0.05 between group I and groups II and III.

**Table 2:** Doppler features of the study groups.

Variable	Group I (IUGR) (*n* = 15)	Group II (AGA with MH) (*n* = 12)	Group III (AGA without MH) (*n* = 13)	*p* value
FO pulsatility index	3.7 ± 0.99	2.84 ± 0.69	2.77 ± 0.44	0.004^∗^
UtA resistance index	0.59 ± 0.16	0.51 ± 0.12	0.49 ± 0.07	0.119
UA resistance index	0.72 ± 0.19	0.61 ± 0.07	0.63 ± 0.11	0.068
MCA resistance index	0.75 ± 0.09	0.76 ± 0.11	0.84 ± 0.09	0.039^‡^

IUGR: intrauterine growth restriction; AGA: adequate for gestational age; MH: maternal hypertension; FO: foramen ovale; UtA: uterine artery; UA: umbilical artery; MCA: median cerebral artery; ^∗^*p* < 0.05 between group I and groups II and III; ^‡^*p* < 0.05 between group III and groups I and II.
