# Corrigendum to “Behaviour of the Foramen Ovale Flow in Fetuses with Intrauterine Growth Restriction”

**DOI:** 10.1155/2018/3725843

**Published:** 2018-07-22

**Authors:** Ângela R. L. Nader, Paulo Zielinsky, Alexandre Antonio Naujorks, Luiz Henrique S. Nicoloso, Antonio Luiz Piccoli Junior, Natássia Miranda Sulis, Luiza Ferreira van der Sand, Victoria de Bittencourt Antunes, Gabriela dos Santos Marinho, Fernanda Greinert dos Santos, Natan Pereira Gosmann, Eduardo Becker Júnior, Renato Frajndlich, Tamara Beherens, Marcelo Brandão da Silva, Carolina Barbisan, Stefano Busato, Mauro Lopes, Caroline Klein

**Affiliations:** ^1^Unidade de Cardiologia Fetal, Instituto de Cardiologia do Rio Grande do Sul, Porto Alegre, RS, Brazil; ^2^Departmento de Pediatria, Universidade Federal do Rio Grande do Sul (UFRGS), Porto Alegre, RS, Brazil; ^3^Hospital de Clinicas de Porto Alegre, Porto Alegre, RS, Brazil

In the article titled “Behaviour of the Foramen Ovale Flow in Fetuses with Intrauterine Growth Restriction” [1], the name of sixteenth author was given incorrectly as Caroline Barbisan. The author's name should have been written as Carolina Barbisan. The revised authors' list is shown above.
